# Performance of NEWS2, RETTS, clinical judgment and the Predict Sepsis screening tools with respect to identification of sepsis among ambulance patients with suspected infection: a prospective cohort study

**DOI:** 10.1186/s13049-021-00958-3

**Published:** 2021-09-30

**Authors:** Ulrika M. Wallgren, Jan Sjölin, Hans Järnbert-Pettersson, Lisa Kurland

**Affiliations:** 1 Department of Clinical Science and Education, Karolinska Institutet, Södersjukhuset, Sjukhusbacken 10, 118 83 Stockholm, Sweden; 2 Fisksätra Vårdcentral (Primary Health Care Center), Fisksätra torg 20, 133 41 Saltsjöbaden, Sweden; 3 Department of Medical Sciences, Akademiska Sjukhuset, Uppsala University, 751 85 Uppsala, Sweden; 4 Department of Medical Sciences, Örebro University, Campus USÖ, Södra Grev Rosengatan 32, 701 12 Örebro, Sweden

**Keywords:** Sepsis, Screening, Emergency medical services, Prehospital, Emergency care

## Abstract

**Background:**

There is little evidence of which sepsis screening tool to use in the ambulance setting. The primary aim of the current study was to compare the performance of NEWS2 (National Early Warning score 2) and RETTS (Rapid Emergency Triage and Treatment System) with respect to identification of sepsis among ambulance patients with clinically suspected infection. The secondary aim was to compare the performance of the novel Predict Sepsis screening tools with that of NEWS2, RETTS and clinical judgment.

**Methods:**

Prospective cohort study of 323 adult ambulance patients with clinically suspected infection, transported to hospitals in Stockholm, during 2017/2018. The sensitivity, specificity, and AUC (Area Under the receiver operating Curve) were calculated and compared by using McNemar´s test and DeLong’s test.

**Results:**

The prevalence of sepsis in the current study population was 44.6% (144 of 323 patients). No significant difference in AUC was demonstrated between NEWS2 ≥ 5 and RETTS ≥ orange. NEWS2 ≥ 7 demonstrated a significantly greater AUC than RETTS red. The Predict Sepsis screening tools ≥ 2 demonstrated the highest sensitivity (range 0.87–0.91), along with RETTS ≥ orange (0.83), but the lowest specificity (range 0.39–0.49). The AUC of NEWS2 (0.73) and the Predict Sepsis screening tools (range 0.75–0.77) was similar.

**Conclusions:**

The results indicate that NEWS2 could be the better alternative for sepsis identification in the ambulance, as compared to RETTS. The Predict Sepsis screening tools demonstrated a high sensitivity and AUCs similar to that of NEWS2. However, these results need to be interpreted with caution as the Predict Sepsis screening tools require external validation.

*Trial registration*: ClinicalTrials.gov, NCT03249597. Registered 15 August 2017—Retrospectively registered, https://clinicaltrials.gov/ct2/show/NCT03249597.

**Supplementary Information:**

The online version contains supplementary material available at 10.1186/s13049-021-00958-3.

## Background

Sepsis is one of the most common medical emergencies and the mortality is high [1–3]. Sepsis is, however, often not identified in a timely fashion [4–6] despite the knowledge that time to treatment is related to patient outcome [7–10]. Time to treatment has been shown to be halved when sepsis is identified in the ambulance [11]. Hence, identification of patients likely to develop sepsis in this setting is important as more than half the patients with sepsis arrive to hospital by ambulance [12].

Screening tools have been shown to increase sepsis identification as compared to clinical judgment [5, 6], but there are a few screening tools developed explicitly for the identification of sepsis in the ambulance [13–19]. Neither the National Early Warning score (NEWS2) [20] nor the rapid emergency triage and treatment system (RETTS) [21, 22], an early warning score and a triage system respectively [20], are initially designed to identify sepsis. The use of NEWS2 is increasing worldwide [20]. It has been implemented in most hospital wards in addition to some emergency departments (EDs) and is gaining interest with some of the ambulance services in Sweden. RETTS is a triage system initially developed in Sweden [23] and is currently the most used triage system both in the ambulance and EDs. Both NEWS2 and RETTS have been proposed to be used to identify sepsis among patients with suspected infection [20, 24, 25], while NEWS2 has been shown to be superior to RETTS in the ED setting [22]. Neither NEWS2 nor RETTS have previously been validated with respect to sepsis identification in the ambulance.

Both NEWS2 and RETTS are based primarily on vital signs. However, more than one third of the patients with severe infection present with normal vital signs [26]. This suggests that including variables other than vital signs is needed for sepsis screening which was also the rationale for the development of the Predict Sepsis screening tools [27]. These tools are unique in that they were developed explicitly for sepsis identification in the ambulance and the result of a prospective, stepwise approach where the association with sepsis was calculated for each variable measured in the ambulance-also including symptoms.

The primary aim of the current study was to compare the performance of NEWS2 and RETTS with respect to identification of sepsis among ambulance patients with clinically suspected infection. The secondary aim was to compare the performance of the novel Predict Sepsis screening tools with that of NEWS2, RETTS and clinical judgment.

## Methods

### Study design and setting

The study was a prospective cohort study of 323 adult non-trauma, ambulance patients with clinically suspected infection transported to hospitals in Stockholm. We compared the performance of NEWS2 and RETTS for the identification of sepsis. Furthermore, the performance of the recently developed Predict Sepsis screening tools was compared with that of NEWS2, RETTS and clinical judgment. The current study was part of the Predict Sepsis study [27] (Clinical Trials identifier NCT03249597).

### Selection of participants

Inclusion criteria were adult (≥ 18 years) non-trauma, ambulance patients, considered to suffer from a new onset infection according to clinical judgment by the ambulance personnel, and required data to determine the outcome sepsis/no sepsis. For details, see Predict Sepsis study [27].

All patients were enrolled by the ambulance personnel during the period of April 3rd, 2017 and August 30th, 2018 and transported by the ambulance provider Samariten Ambulans AB [28] to one of the seven major hospital EDs in Stockholm city county [27]. All ambulances were staffed with at least one nurse specialist and one emergency medical technician [29].

The exclusion criterium was participants lacking data required to complete each screening model.

### Definition of outcomes

#### Sepsis

Sepsis was defined in accordance with the Sepsis-3 criteria [30], i.e., infection [6, 27] in combination with an increased SOFA score of ≥ 2 points, within 36 h from ED arrival [27]. The preexisting score was set to zero for patients with no previous recording of baseline data [27, 30]. Septic shock was defined as sepsis in combination with indication for vasopressor treatment and a serum lactate level greater than 2 mmol/L within 36 h from ED arrival [27, 30].

#### No sepsis

“No sepsis” was defined as not fulfilling above criteria for sepsis.

### Sepsis screening models

NEWS2 (described in Table 1) is the 2017 updated version of NEWS, originally designed by the Royal College of Physicians in 2012 and it is based on six vital signs [20]. A NEWS2 score of 5 or more is used as indicative of potential serious acute clinical deterioration and the need for an urgent response [20]. A NEWS2 score of 7 or more is considered indicative of a severely ill patient, in need of an emergency response including personnel with critical care competence [20, 31].

**Table 1:** NEWS2, RETTS and the Predict Sepsis screening tools

	NEWS2 (20)	RETTS (21)	Predict Sepsis screening tool 1 (27)	Predict Sepsis screening tool 2 (27)	Predict Sepsis screening tool 3 (27)
Number of included variables	6	9 variables in VS part described below + ESS*	6	4	6
Score considered positive for suspected sepsis	≥ 5 is recom-mended (20) but some sepsis alerts (35) suggest ≥ 7	RETTS red is used in some sepsis alerts (36)	≥ 2	≥ 2	≥ 2
*Included variables with weights*					
Respiratory rate	≤ 8 = 3 9–11 = 1 12–20 = 0 21–24 = 2 ≥ 25 = 3	> 30 or < 8 = red 26–30 = orange 8–25 = green	X	X	> 24 = 1
Oxygen saturation	≤ 91 = 3 92–93 = 2 94–95 = 1 ≥ 96 = 0	< 90 with O2 = red < 90 without O2 = orange 90–95 without O2 = yellow > 95 without O2 = green	X	X	< 94 = 1
Systolic blood pressure	≤ 90 = 3 91–100 = 2 101–110 = 1 111–219 = 0 ≥ 220 = 3	< 90 = red	≤ 100 = 2	≤ 100 = 2	≤ 100 = 2
Heart rate	≤ 40 = 3 41–50 = 1 51–90 = 0 91–110 = 1 111–130 = 2 ≥ 131 = 3	Regular > 130 or irregular > 150 = red > 120 or < 40 = orange > 110 or < 50 = yellow 50–110 = green	X	X	> 110 = 1
Consciousness	(AVPU in parenthesis) GCS ≤ 14 (C, V, P, or U) = 3 GCS ≥ 15 (A) = 0	(RLS in parenthesis) GCS < 8 (unconscious) = red GCS 8–12 (2–3) = orange GCS 13–14 (acutely disoriented) = yellow GCS 15 (alert) = green	GCS < 15 = 2 History of acute altered mental status = 1	History of acute altered mental status and/or GCS < 15 = 1	GCS < 15 = 2
Temperature, degrees C	≤ 35,0 = 3 35,1–36,0 = 1 36,1–38,0 = 0 38,1–39,0 = 1 ≥ 39,1 = 2	> 41 or < 35 = orange 38.6–41 = yellow 35–38.5 = green	38.1–38.5 = 1 > 38.5 = 2	38.1–38.5 = 1 > 38.5 = 2	38.1–38.5 = 1 > 38.5 = 2
Other variables	X	Obstructed airway = red Stridor = red Ongoing seizures = red	Gastrointestinal symptoms = 1 P-Lactate > 4.0 = 2	Gastrointestinal symptoms = 1	X

NEWS2, National Early Warning Score 2; RETTS, Rapid Emergency Triage and Treatment System; VS, Vital sign; ESS, Emergency Symptoms and Signs; GCS, Glasgow Coma Scale; AVPU, Swedish consciousness scale (Alert, Verbal responsive, Pain responsive, Unresponsive); C, Confusion; V, response to Voice; P, response to Pain; U, Unresponsive

RETTS [21] is a triage system developed and licensed by Predicare AB [23]. It is a five-graded color scale, based on vital signs (VS, see Table 1 for a description) and Emergency Symptoms and Signs (ESS) which reflect presentation and symptoms. The most pronounced vital sign or ESS deviation will decide the triage level. Red is the highest triage level (defined as life threatening), followed by orange (potentially life threatening), yellow, green, and blue [23].

Sepsis, according to clinical judgment, was defined as the primary assessed condition sepsis (code C05) as recorded in the ambulance record.

The Predict Sepsis screening tools [27] are presented in Table 1. The Predict Sepsis screening tool 1 is based on symptoms, vital signs, and lactate. Predict Sepsis screening tool 2 is based on four variables of which two are vital signs and two are symptom-based. Predict Sepsis screening tool 3 is based on vital signs alone, but with novel cut-offs calculated to have the strongest association with the outcome sepsis.

### Measurements; data collection and handling

Eight keywords related to medical history (“fever or suspected fever”, “pain”, “acute altered mental status”, “weakness of the legs”, “breathing difficulties”, “loss of energy”, “gastrointestinal symptoms” and “risk factors for sepsis”) and six vital signs (respiratory rate, oxygen saturation, heart rate, systolic blood pressure, Glasgow coma scale; GCS and temperature) were collected through a Case Report Form (CRF) used in the ambulance as part of the Predict sepsis study [27]. Priority level according to RETTS, vital signs not recorded in the CRF and primary assessed condition were extracted from the ambulance records (amPHI® Prehospital ambulance record, Amphi Systems A/S, Aalborg, Denmark, through the hospital medical record TakeCare®, v. 18.3.10, CompuGroup Medical, Stockholm, Sweden) and the local digital IT-support for prehospital care in Stockholm; FRAPP® (Framtida IT-plattform för prehospital vård i Stockholms läns landsting).

Data related to ED arrival time, age, gender, criteria for suspicion of a new-onset infection included in the Sepsis-3 definition of sepsis, in-hospital vital signs/ laboratory tests/ mortality and discharge International Classification of Diseases (ICD) code were extracted from the hospital medical records [27].

### Statistical analyses

Statistical analyses were performed using SPSS (Statistical Package for the Social Sciences) version 27.0 (SPSS Inc., Chicago, IL, USA), and Clinical Research Calculators; Calculator 1, Vassarstats.net [32].

Sensitivity, specificity, positive predictive value (PPV), negative predictive value (NPV) and likelihood ratio (positive and negative LR) of NEWS2, RETTS, clinical judgment and the three Predict Sepsis screening tools were calculated in relation to outcome sepsis and outcome septic shock by Vassarstat.net, Clinical Calculator 1 [32]. The area under the receiver operating curve (AUC) was calculated (using SPSS) for the models without cut-offs (based on sum of scores) and with specific cut-offs. The sensitivity and specificity of each model for the outcomes sepsis and septic shock were compared using McNemar´s test. The AUC for the outcomes sepsis and septic shock was compared using DeLong´s test. *P*-values ˂0.05 were considered statistically significant.

### Ethical approval

The study was approved by the Stockholm Regional Ethical Review Board (reference number 2016/2001-31/2, 2018/2202 and 2020-03894). Written consent was obtained from all participants.

The current study complied with the Declaration of Helsinki [33] and the manuscript was drafted according to the Standards for the Reporting of Diagnostic accuracy studies (STARD) criteria [34].

## Results

### Characteristics

551 patients with clinically suspected infection were included in the Predict Sepsis study [27]. The 323 patients that had the data required to complete each screening model were included in the current study. Of these, 144 (44.6%) had sepsis.

For characteristics of the study participants, see Table 2. Fifteen out of 144 septic patients (10.4%) died during in-hospital stay. The highest in-hospital mortality was observed among patients with RETTS red (7/48 patients, 14.6%) or NEWS2 ≥ 7 (15/123 patients, 12.2%), see Additional file 1.

**Table 2:** Characteristics of the 323 ambulance patients with suspected infection and the 144 patients with sepsis

Variable	All patients		Patients with outcome sepsis	
	Number* (%*)	Median (IQR)	Number* (%*)	Median (IQR)
Age (yr)		78 (72–85)		78 (70–84)
< 65 years	36/323 (11.1)		18/144 (12.5)	
65–74 years	78/323 (24.1)		39/144 (27.1)	
≥ 75 years	209/323 (64.7)		87/144 (60.4)	
*Gender*				
Male	189/323 (58.5)		90/144 (62.5)	
*Ambulance priority*				
1	62/320 (19.4)		48/143 (33.6)	
2	222/320 (69.4)		88/143 (61.5)	
3	36/320 (11.3)		7/143 (4.9)	
Admitted to in-hospital care	273/323 (84.5)		139/144 (96.5)	
*Outcome*				
Sepsis	144/323 (44.6)			
No sepsis	179/323 (55.4)			
*Septic shock*^a^			17/138 (12.3)	
*ICD-code upon hospital discharge*				
ICD-code sepsis	29/321 (9.0)		24/142 (16.9)	
ICD-code infection	211/321 (65.7)		118/142 (83.1)	
In-hospital mortality	21/323 (6.5)		15/144 (10.4)	

IQR, Interquartile range; ICD, International Statistical Classification of Diseases and Related Health Problems

*Of patients with documented variable

^a^septic shock defined in accordance with Sepsis-3 within 36 h from emergency department arrival

### Performance of the screening models

**Fig. 1:**
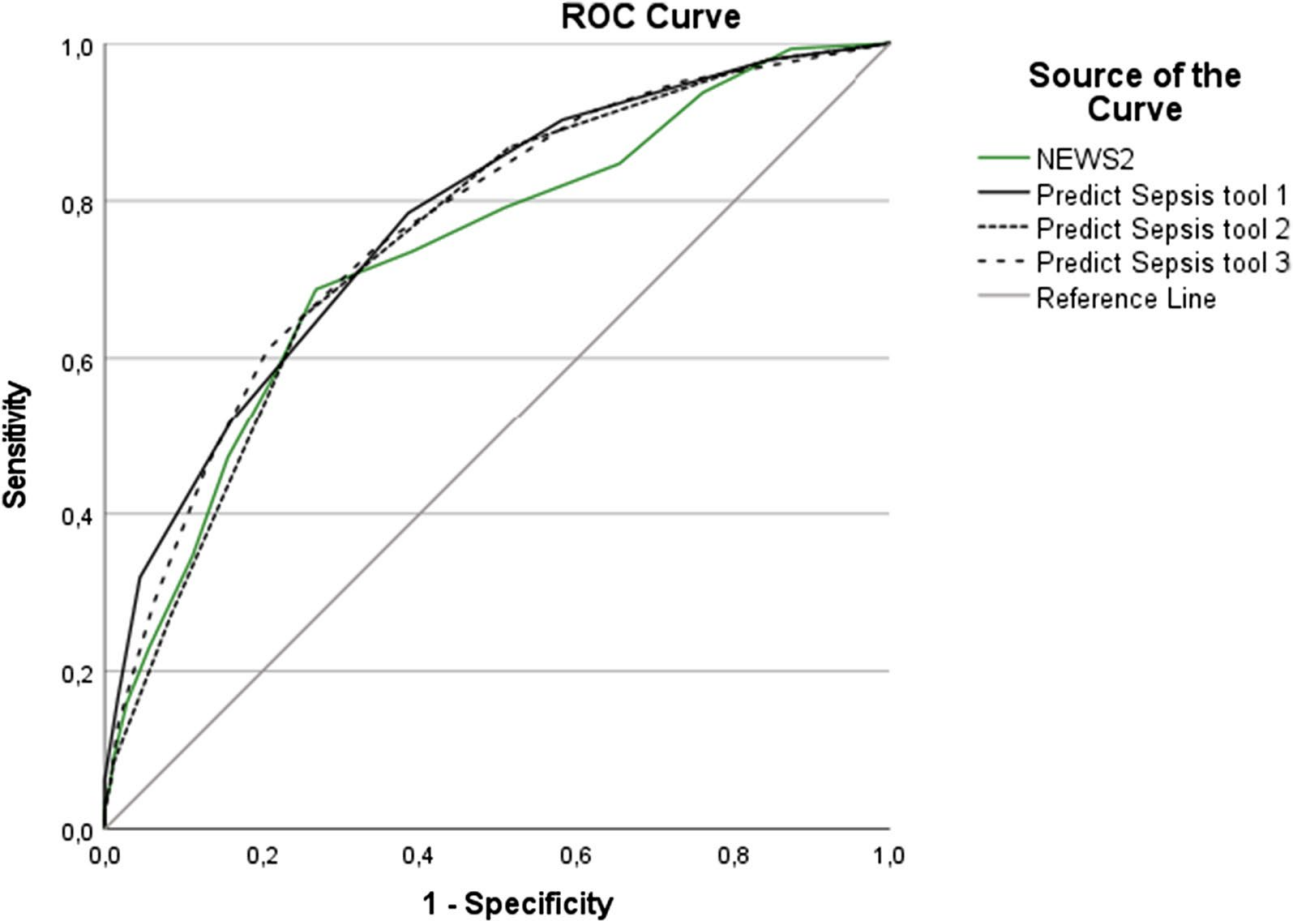
ROC curves for models without cut-offs and sepsis*. ROC, Receiver Operating Characteristic Curve; NEWS2, National Early Warning Score 2; RETTS, Rapid Emergency Triage and Treatment System. *ROC curves based on sum of scores for NEWS2 and the Predict Sepsis screening tools, with respect to outcome sepsis

**Fig. 2:**
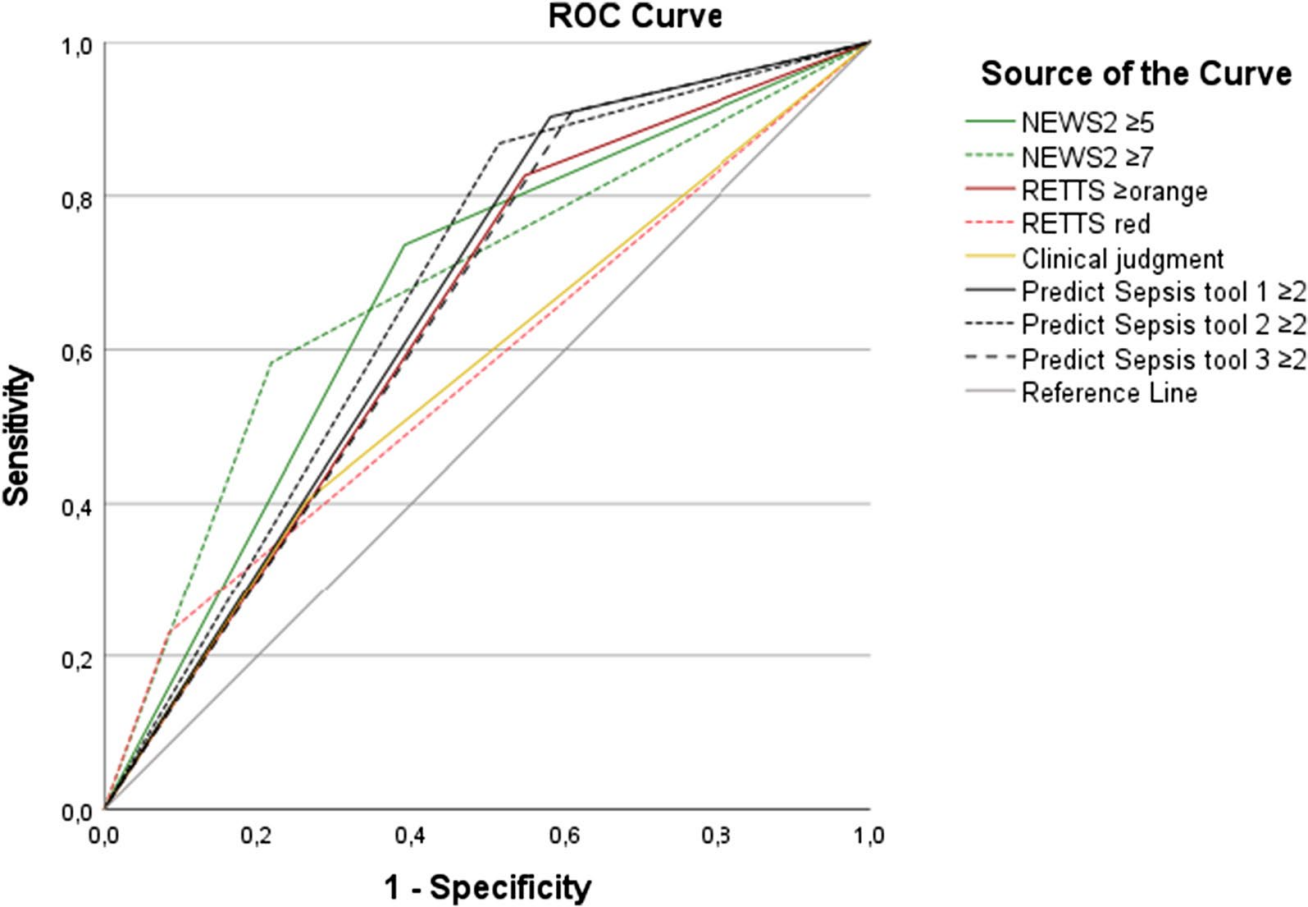
ROC curves for models with specific cut-offs and sepsis*. ROC, Receiver Operating Characteristic Curve; NEWS2, National Early Warning Score 2; RETTS, Rapid Emergency Triage and Treatment System. *ROC curves for all the models, with specific cut-offs, with respect to outcome sepsis

See Table 3 for the performance of NEWS2, RETTS, clinical judgment and the Predict Sepsis tools with respect to sepsis identification, and Additional file 2–3 for McNemar´s test for comparison of sensitivity and specificity, Additional file 4–5 for DeLong’s test for comparison of AUC for the models with and without specific cut-offs and Figs. 1 and 2 for the Receiver Operating Characteristics; ROC curves.

**Table 3:** Performance of the screening models with respect to identification of sepsis

	NEWS2 (20) ≥ 5	NEWS2 ≥ 7	RETTS (21) ≥ orange	RETTS red	Clinical judgment	Predict Sepsis screening tool 1 (27)	Predict Sepsis screening tool 2 (27)	Predict Sepsis screening tool 3 (27)
Total score considered positive for predicted sepsis	≥ 5	≥ 7	RETTS ≥ orange	RETTS red	Clinical judgment sepsis by ambulance personnel	≥ 2	≥ 2	≥ 2
Sensitivity^a^ (95%CI)	0.74 (0.65–0.80)	0.58 (0.50–0.66)	0.83 (0.75–0.88)	0.23 (0.17–0.31)	0.40 (0.32–0.49)	0.90 (0.84–0.94)	0.87 (0.80–0.92)	0.91 (0.85–0.95)
Specificity^a^ (95%CI)	0.61 (0.53–0.68)	0.78 (0.71–0.84)	0.45 (0.38–0.53)	0.92 (0.86–0.95)	0.74 (0.67–0.80)	0.42 (0.35–0.50)	0.49 (0.41–0.56)	0.39 (0.32–0.47)
PPV^a^ (95%CI)	0.60 (0.53–0.67)	0.68 (0.59–0.76)	0.55 (0.48–0.62)	0.69 (0.54–0.81)	0.55 (0.45–0.65)	0.56 (0.49–0.62)	0.58 (0.51–0.64)	0.55 (0.48–0.61)
NPV^a^ (95%CI)	0.74 (0.66–0.81)	0.70 (0.63–0.76)	0.76 (0.67–0.84)	0.60 (0.54–0.65)	0.61 (0.54–0.67)	0.84 (0.75–0.91)	0.82 (0.73–0.89)	0.84 (0.74–0.91)
Pos LR^a^ (95%CI)	1.88 (1.53–2.32)	2.68 (1.96–3.65)	1.51 (1.30–1.76)	2.73 (1.55–4.83)	1.53 (1.12–2.10)	1.55 (1.36–1.78)	1.69 (1.44–1.97)	1.49 (1.31–1.70)
Neg LR^a^ (95%CI)	0.43 (0.33–0.57)	0.53 (0.44–0.65)	0.38 (0.27–0.56)	0.84 (0.77–0.92)	0.81 (0.71–0.93)	0.23 (0.14–0.39)	0.27 (0.18–0.42)	0.23 (0.14–0.39)
AUC^a^ for the model without cut-off-based on sum of scores (95%CI)	0.73 (0.68–0.79)	0.73 (0.68–0.79)	not possible to calculate	not possible to calculate	not possible to calculate	0.77 (0.72–0.82)	0.75 (0.70–0.80)	0.77 (0.71–0.82)
AUC^a^ for the model with specific cut–off	0.67 (0.61–0.73)	0.68 (0.62–0.74)	0.64 (0.58–0.70)	0.57 (0.51–0.64)	0.57 (0.51–0.63)	0.66 (0.60–0.72)	0.68 (0.62–0.74)	0.65 (0.59–0.71)

For pairway comparisons of sensitivity, specificity, and AUC-values between the models, for outcome sepsis, see Additional file 2–5

NEWS2, National Early Warning score 2; RETTS, Rapid Emergency Triage and Treatment System; CI, Confidence Interval; PPV, Positive Predictive Value; NPV, Negative Predictive Value; LR, Likelihood Ratio; AUC, Area Under the (receiver operating) Curve

^a^With respect to outcome sepsis within 36 h from emergency department arrival, among ambulance patients with clinically suspected infection

#### NEWS2 compared to RETTS

No significant difference in performance was demonstrated when NEWS2 ≥ 5 and RETTS ≥ orange were compared (Table 3 and Additional file 5).

NEWS2 ≥ 7 demonstrated a significantly higher AUC than RETTS red (*P*-value < 0.001, see Table 3 and Additional file 5).

#### Predict Sepsis screening tools compared to NEWS2, RETTS and clinical judgment with respect to outcome sepsis

The Predict Sepsis screening tools demonstrated a significantly higher sensitivity (ranging between 0.87 and 0.91) and lower specificity (ranging between 0.39 and 0.49) as compared to NEWS2 (≥ 5 and ≥ 7), RETTS red and clinical judgment (see Table 3, Additional file 2, 3).

The AUC (based on sum of scores) of the Predict Sepsis screening tools (tool 1: 0.77, tool 2: 0.75, tool 3: 0.77) was similar to that of NEWS2 (0.73) (see Table 3, Additional file 4).

RETTS red demonstrated a significantly lower sensitivity (0.23) than all the other screening models except for clinical judgment (Table 3 and Additional file 2).

The specificity of RETTS red (0.92) was higher than that of all the other models (Table 3, Additional file 3).

### Additional findings; comparison of performance of the screening models with respect to identification of septic shock

See Additional file 6–12 for the performance of NEWS2, RETTS, clinical judgment and the Predict Sepsis tools, with respect to identification of septic shock.

The Predict Sepsis screening tool based solely on vital signs (tool 3) and RETTS ≥ orange identified 17/17 patients (100.0%) that developed septic shock (Additional file 6). RETTS red identified a significantly lower proportion of patients (8/17 patients, 47.1%) that developed septic shock, as compared to all screening models except for clinical judgment that identified 13/17 patients, 76%, see Additional file 6–7.

## Discussion

This is the first prospective study to compare the performance of NEWS2 and RETTS in the ambulance setting for the identification of sepsis. The results of the current study indicated no major difference with respect to sepsis identification when based on comparisons of the AUC of RETTS orange, NEWS2 (both NEWS2 ≥ 5 and NEWS2 ≥ 7) and the Predict Sepsis screening tools. However, RETTS red and clinical judgment demonstrated a significantly lower AUC as compared to the other models with respect to sepsis. The Predict Sepsis screening tools showed promising results of a high sensitivity but, conversely, a low specificity.

### The performance of the screening models

A NEWS2 score of 5 or more identified three of four septic patients and nine of ten patients who developed septic shock. The Royal College of Physicians recommends a NEWS2 score of 5 or above to be considered as suspected sepsis among patients with clinical suspicion of infection and recommend a rapid escalation of clinical care in addition to urgent treatment for these patients [20]. There is an ongoing discussion [35] to apply a NEWS2 cut-off of 7 or higher to identify the sickest septic patients. This cut-off is supported by the results of the current study showing that eight of ten patients who developed septic shock were identified.

RETTS highest priority level (i.e., “red”) appears to be insufficient for sepsis identification due to the low sensitivity for sepsis. The low sensitivity may be explained by the cut-off for respiratory rate being high while that for GCS require an unconscious patient, resulting in a lower proportion of patients fulfilling these criteria. RETTS red has been suggested to be used to identify patients with severe sepsis and septic shock [36]. However, it failed to identify more than half of the patients who developed septic shock in the current study. A better alternative would be to use the second triage level, i.e., RETTS ≥ orange, which identified four of five septic patients and all patients who developed septic shock.

Four of ten patients that developed sepsis were identified by clinical judgment which was higher than previously demonstrated [5, 11]. Enhanced attention on sepsis, including clinical updates of the Swedish ambulance guidelines [37], may have contributed to these results. Additionally, sepsis awareness among ambulance personnel was likely to have been affected by the Predict Sepsis study itself. Nonetheless, the current results support that applying a screening tool increases sepsis identification.

The Predict Sepsis screening tools, of which the two first tools include symptom variables, demonstrated the highest sensitivity, together with RETTS orange, but a low specificity and the AUCs were similar to that of NEWS2. The major disadvantage of these tools was the low specificity. The Predict Sepsis tools did however capture almost all the patients who developed septic shock.

### The choice of a screening tool; sensitivity versus specificity

It is a well described challenge to development of a screening tool combining both a high sensitivity and a high specificity. A low specificity may cause false sepsis alerts leading to an over-use of resources, while a low sensitivity may lead to missing septic patients resulting in an increased mortality and morbidity. We advocate that screening tools should have a high sensitivity and that false sepsis alerts could be reduced by the assessment of an experienced clinician after the initial screening, since the specificity of experienced clinicians has been shown to be high [6]. In our opinion, the screening model should be regarded as a first step in the clinical decision process that leads to a correct diagnosis.

The timing of the identification and treatment of septic patients without septic shock has been questioned [38, 39]. Nevertheless, we believe that all septic patients benefit from early identification as this not only allows for early treatment, but also enables monitoring of the patient from an early stage of care. Moreover, international guidelines, such as the Surviving Sepsis Campaign, recommend treatment within 1 h from the identification of all septic patients, not only for those suffering from septic shock [40].

### Strengths and limitations of the current study

This is the first prospective study to compare the performance of NEWS2 and RETTS in the ambulance setting for the identification of sepsis, which is considered a strength of the study.

There are several limitations to the current study.

First, the Predict Sepsis screening tools were compared to NEWS2, RETTS and clinical judgment in the same population in which the Predict Sepsis tools were developed. This infers a risk of over-adapting the new model to the data material from where it was derived. Hence, the discriminative properties of the Predict Sepsis screening tools may be lower in another population and an external validation of the Predict Sepsis screening tools is therefore needed.

Second, calculation of the AUC based on sum of scores was not possible for RETTS since vital signs were registered but not the ESS data. However, all RETTS levels include information on ESS to decide the documented priority level and accordingly the calculated sensitivity and specificity are considered to be correct. Additionally, the AUC of RETTS with specific cut-offs was calculated and compared to that of the other models, in turn also given specific cut-offs.

Third, the results are based on the study population, i.e., patients with a suspected infection and are therefore not generalizable to the general ambulance population. Ideally, a sample representative of “all” ambulance patients should have been included for a screening tool to be applicable to the general ambulance population. This would have enabled study of the identification of patients that are not easily recognized as having an infection, e.g., the elderly with non-specific symptoms and those lacking fever. The inclusion of a sample of general ambulance patients was, however, not feasible at the time but would be of value in future studies.

Forth, the current study is the second part of the larger Predict Sepsis study [27]. The original power calculation was performed for the purpose of including enough patients with the outcome sepsis in relation to variables studied for the association with sepsis, and to develop the Predict Sepsis screening tools. Hence, the power calculation was not performed explicitly for the current study. However, we believe the results of comparing the performance of the screening models, also those in clinical use, in this study of prospectively included ambulance patients are of interest.

Finally, the Predict Sepsis study was not designed for the outcome septic shock and the number of patients who developed septic shock was small. The results relating to the performance of identifying septic shock should therefore be interpreted with caution and repeated in larger studies.

## Conclusions

The results indicate that NEWS2 may be a better alternative than RETTS with respect to the identification of sepsis among patients with suspected infection in the ambulance setting. This conclusion is based on the results indicating that there is no difference between NEWS2 and RETTS when comparing the second highest priority levels, but a superior performance of NEWS2 when comparing the highest priority levels. The Predict Sepsis screening tools showed promising results with respect to a high sensitivity for sepsis and the AUCs were similar to that of NEWS2. However, these results need to be interpreted with caution as the Predict Sepsis screening tools require external validation.

## Supplementary Information

**Additional file 1**. Observed in-hospital mortality for patients identified as septic by the models.

**Additional file 2**. McNemar's test, pairwise comparison of sensitivity for sepsis.

**Additional file 3**. McNemar's test, pairwise comparison of specificity for sepsis.

**Additional file 4**. DeLong's test, pairwise comparison of AUC for sepsis for models without cut-offs.

**Additional file 5**. DeLong's test, pairwise comparison of AUC for sepsis for models with specific cut-offs.

**Additional file 6**. Performance of the screening models with respect to identification of septic shock.

**Additional file 7**. McNemar's test, pairwise comparison of sensitivity for septic shock.

**Additional file 8**. McNemar's test, pairwise comparison of specificity for septic shock.

**Additional file 9**. DeLong's test, pairwise comparison of AUC for septic shock for models without cut-offs.

**Additional file 10**. DeLong's test, pairwise comparison of AUC for septic shock for models with specific cut-offs.

**Additional file 11**. ROC curves for models without cut-offs and septic shock.

**Additional file 12**. ROC curves for models with specific cut-offs and septic shock.

## Data Availability

The data that support the findings of this study are available from Karolinska Institutet Södersjukhuset but restrictions apply to the availability of these data, which were used under license for the current study, and are not publicly available. Data are however available from the authors upon reasonable request and with permission of Karolinska Institutet Södersjukhuset.

## Abbreviations

NEWS2: National Early Warning Score 2
RETTS: Rapid Emergency Triage and Treatment System
AUC: Area Under the receiver operating Curve
NCT: National Clinical Registration
ED: Emergency Department
ESS: Emergency Symptoms and Signs
GCS: Glasgow Coma Scale
CRF: Case Report Form
FRAPP: Framtida IT-plattform för prehospital vård i Stockholms läns landsting (“future Information Technology platform for prehospital care in Stockholm City Council”—the name of the ambulance medical record system of Stockholm)”
ICD: International Classification of Diseases
SPSS: Statistical Package for the Social Sciences
PPV: Positive Predictive Value
NPV: Negative Predictive Value
LR: Likelihood Ratio
STARD: Standards for the reporting of diagnostic accuracy studies
ROC: Receiver Operating Characteristic
IQR: Interquartile range
