# Supplementary material for: Performance of NEWS2, RETTS, clinical judgment and the Predict Sepsis screening tools with respect to identification of sepsis among ambulance patients with suspected infection: a prospective cohort study
Source: Scand J Trauma Resusc Emerg Med. 2021 Sep 30;29:144. doi: 10.1186/s13049-021-00958-3 (PMC8485465; doi:10.1186/s13049-021-00958-3)
Supplement: Supplementary file 1 — Additional file 1. Observed in-hospital mortality for patients identified as septic by the models. [file 13049_2021_958_MOESM1_ESM.pdf]

Additional file 1. Observed in-hospital mortality for patients identified as septic by the models.

	NEWS2[1] ≥5	NEWS2 ≥7	RETTS[2] ≥orange	RETTS red	Clinical judgment	Predict Sepsis tool 1[3]	Predict Sepsis tool 2[3]	Predict Sepsis tool 3[3]
In-hospital mortality for patients identified as septic by the model (%)	17/176 (9.7)	15/123 (12.2)	16/217 (7.4)	7/48 (14.6)	6/105 (5.7)	18/234 (7.7)	16/217 (7.4)	17/240 (7.1)

NEWS2=National Early Warning score 2, RETTS= Rapid Emergency Triage and Treatment System.

References:
1) Royal College of Physicians. National Early Warning Score (NEWS) 2- Standardising the assessment of acute-illness severity in the NHS, Updated report of a working party December 2017.
2) Widgren BR, Jourak M. Medical Emergency Triage and Treatment System (METTS): a new protocol in primary triage and secondary priority decision in emergency medicine. The Journal of emergency medicine. 2011.
3) Wallgren UM, Sjölin J, Järnbert-Pettersson H, Kurland L. The predictive value of variables measurable in the ambulance and the development of the Predict Sepsis screening tools: a prospective cohort study. Scandinavian journal of trauma, resuscitation and emergency medicine. 2020.
