# Supplementary material for: Performance of NEWS2, RETTS, clinical judgment and the Predict Sepsis screening tools with respect to identification of sepsis among ambulance patients with suspected infection: a prospective cohort study
Source: Scand J Trauma Resusc Emerg Med. 2021 Sep 30;29:144. doi: 10.1186/s13049-021-00958-3 (PMC8485465; doi:10.1186/s13049-021-00958-3)
Supplement: Supplementary file 2 — Additional file 2. McNemar's test, pairwise comparison of sensitivity for sepsis. [file 13049_2021_958_MOESM2_ESM.pdf]

Additional file 2. McNemar´s test, pairwise comparison of sensitivity for sepsis.

Compared with:	**NEWS2[1] ≥5**	**NEWS2 ≥7**	**RETTS[2] ≥orange**	**RETTS red**	**Clinical Judgment**	**Predict Sepsis tool 1[3] ≥2**	**Predict Sepsis tool 2[3] ≥2**	**Predict Sepsis tool 3[3] ≥2**
**NEWS2 ≥5**	X							
**NEWS2 ≥7**	**<0.001**	X						
**RETTS ≥orange**	**0.019**	**<0.001**	X					
**RETTS red**	**<0.001**	**<0.001**	**<0.001**	X				
**Clinical Judgment**	**<0.001**	**0.001**	**<0.001**	**0.001**	X			
**Predict Sepsis tool 1 ≥2**	**<0.001**	**<0.001**	0.052	**<0.001**	**<0.001**	X		
**Predict Sepsis tool 2 ≥2**	**0.004**	**<0.001**	0.362	**<0.001**	**<0.001**	0.063	X	
**Predict Sepsis tool 3 ≥2**	**<0.001**	**<0.001**	**0.008**	**<0.001**	**<0.001**	0.999	0.210	X

NEWS2=National Early Warning score 2, RETTS= Rapid Emergency Triage and Treatment System.

P-values derived from McNemar´s test are presented in the table. Bold numbers of P-values indicate a significant difference between the models with respect to sensitivity for sepsis.

References:
1) Royal College of Physicians. National Early Warning Score (NEWS) 2- Standardising the assessment of acute-illness severity in the NHS, Updated report of a working party December 2017.
2) Widgren BR, Jourak M. Medical Emergency Triage and Treatment System (METTS): a new protocol in primary triage and secondary priority decision in emergency medicine. The Journal of emergency medicine. 2011.
3) Wallgren UM, Sjölin J, Järnbert-Pettersson H, Kurland L. The predictive value of variables measurable in the ambulance and the development of the Predict Sepsis screening tools: a prospective cohort study. Scandinavian journal of trauma, resuscitation and emergency medicine. 2020.
