# Supplementary material for: Performance of NEWS2, RETTS, clinical judgment and the Predict Sepsis screening tools with respect to identification of sepsis among ambulance patients with suspected infection: a prospective cohort study
Source: Scand J Trauma Resusc Emerg Med. 2021 Sep 30;29:144. doi: 10.1186/s13049-021-00958-3 (PMC8485465; doi:10.1186/s13049-021-00958-3)
Supplement: Supplementary file 4 — Additional file 4. DeLong's test, pairwise comparison of AUC for sepsis for models without cut-offs. [file 13049_2021_958_MOESM4_ESM.pdf]

Additional file 4. DeLong´s test, pairwise comparison of AUC for sepsis for models without cut-offs.

Compared with:	**NEWS2**[1]	**Predict Sepsis tool 1**[2]	**Predict Sepsis tool 2**[2]	**Predict Sepsis tool 3**[2]
**NEWS2**	X			
**Predict Sepsis tool 1**	0.188	X		
**Predict Sepsis tool 2**	0.610	0.214	X	
**Predict Sepsis tool 3**	**0.010**	0.806	0.562	X

NEWS2=National Early Warning score 2, RETTS= Rapid Emergency Triage and Treatment System.

P-values derived from DeLong´s test are presented in the table. Bold numbers of P-values indicate a significant difference between the AUC values (based on sum of scores) for the models with respect to outcome sepsis.

References:

1) Royal College of Physicians. National Early Warning Score (NEWS) 2- Standardising the assessment of acute-illness severity in the NHS, Updated report of a working party December 2017.
2) Wallgren UM, Sjölin J, Järnbert-Pettersson H, Kurland L. The predictive value of variables measurable in the ambulance and the development of the Predict Sepsis screening tools: a prospective cohort study. Scandinavian journal of trauma, resuscitation and emergency medicine. 2020.
