# Supplementary material for: Performance of NEWS2, RETTS, clinical judgment and the Predict Sepsis screening tools with respect to identification of sepsis among ambulance patients with suspected infection: a prospective cohort study
Source: Scand J Trauma Resusc Emerg Med. 2021 Sep 30;29:144. doi: 10.1186/s13049-021-00958-3 (PMC8485465; doi:10.1186/s13049-021-00958-3)
Supplement: Supplementary file 6 — Additional file 6. Performance of the screening models with respect to identification of septic shock. [file 13049_2021_958_MOESM6_ESM.pdf]

Additional file 6. Performance of the screening models with respect to identification of septic shock.

	**NEWS2[1] ≥5**	**NEWS2 ≥7**	**RETTS[2] ≥orange**	**RETTS red**	**Clinical judgment**	**Predict Sepsis tool 1[3] ≥2**	**Predict Sepsis tool 2[3] ≥2**	**Predict Sepsis tool 3[3] ≥2**
Total score considered positive for predicted sepsis	≥5	≥7	RETTS ≥ orange	RETTS red	Clinical judgment sepsis by ambulance personnel	≥2	≥2	≥2
Sensitivity[α] (95%CI)	0.94 (0.69-1.00)	0.82 (0.56-0.95)	1.00 (0.77-1.00)	0.47 (0.24-0.71)	0.76 (0.50-0.92)	0.94 (0.69-1.00)	0.94 (0.69-1.00)	1.00 (0.77-1.00)
Specificity[α] (95%CI)	0.28 (0.20-0.37)	0.44 (0.35-0.53)	0.20 (0.13-0.28)	0.79 (0.71-0.86)	0.63 (0.54-0.71)	0.09 (0.05-0.16)	0.13 (0.08-0.21)	0.09 (0.05-0.16)
PPV[α] (95%CI)	0.16 (0.09-0.24)	0.17 (0.10-0.27)	0.15 (0.09-0.23)	0.24 (0.12-0.43)	0.22 (0.13-0.36)	0.13 (0.08-0.20)	0.13 (0.08-0.21)	0.13 (0.08-0.21)
NPV[α] (95%CI)	0.97 (0.83-1.00)	0.95 (0.84-0.99)	1.00 (0.83-1.00)	0.91 (0.84-0.96)	0.95 (0.87-0.98)	0.92 (0.60-1.00)	0.94 (0.69-1.00)	1.00 (0.68-1.00)
Pos LR[α] (95%CI)	1.31 (1.11-1.54)	1.47 (1.12-1.92)	1.25 (1.14-1.36)	2.28 (1.23-4.21)	2.06 (1.45-2.92)	1.04 (0.91-1.18)	1.08 (0.95-1.24)	1.10 (1.04-1.16)
Neg LR[α] (95%CI)	0.21 (0.03-1.46)	0.40 (0.14-1.15)	0 (0-NaN)	0.67 (0.42-1.05)	0.37 (0.16-0.89)	0.65 (0.08-5.09)	0.44 (0.06-3.31)	0 (0-NaN)

AUC[α] for the model without cut-off -based on sum of scores	0.69 (0.56-0.82)	0.69 (0.56-0.82)	not possible to calculate	not possible to calculate	not possible to calculate	0.71 (0.56-0.85)	0.64 (0.50-0.78)	0.65 (0.51-0.79)
AUC[α] for the model with specific cut-off	0.61	0.63	0.60	0.63	0.70	0.52	0.54	0.55

NEWS2=National Early Warning score 2, CI=Confidence Interval. NaN=calculation cannot be performed since the values entered include one or more instances of zero.

[α]with respect to outcome septic shock within 36 hours from emergency department arrival, among ambulance patients with clinically suspected infection.

For pairway comparisons of sensitivity, specificity, and AUC-values between the models, with respect to outcome septic shock, see Supplemental Appendix 7-12.

References:
1) Royal College of Physicians. National Early Warning Score (NEWS) 2- Standardising the assessment of acute-illness severity in the NHS, Updated report of a working party December 2017.
2) Widgren BR, Jourak M. Medical Emergency Triage and Treatment System (METTS): a new protocol in primary triage and secondary priority decision in emergency medicine. The Journal of emergency medicine. 2011.
3) Wallgren UM, Sjölin J, Järnbert-Pettersson H, Kurland L. The predictive value of variables measurable in the ambulance and the development of the Predict Sepsis screening tools: a prospective cohort study. Scandinavian journal of trauma, resuscitation and emergency medicine. 2020.
