# Supplementary material for: Performance of NEWS2, RETTS, clinical judgment and the Predict Sepsis screening tools with respect to identification of sepsis among ambulance patients with suspected infection: a prospective cohort study
Source: Scand J Trauma Resusc Emerg Med. 2021 Sep 30;29:144. doi: 10.1186/s13049-021-00958-3 (PMC8485465; doi:10.1186/s13049-021-00958-3)
Supplement: Supplementary file 7 — Additional file 7. McNemar's test, pairwise comparison of sensitivity for septic shock. [file 13049_2021_958_MOESM7_ESM.pdf]

**Additional file 7. McNemar´s test, pairwise comparison of sensitivity for septic shock.**

| | NEWS2[1] ≥5 | NEWS2 ≥7 | RETTS[2] ≥orange | RETTS red | Clinical Judgment | Predict Sepsis tool 1[3] ≥2 | Predict Sepsis tool 2[3] ≥2 | Predict Sepsis tool 3[3] ≥2 |
|---|---|---|---|---|---|---|---|---|
| **NEWS2 ≥5** | X | | | | | | | |
| **NEWS2 ≥7** | 0.500 | X | | | | | | |
| **RETTS ≥orange** | N | N | X | | | | | |
| **RETTS red** | **0.008** | **0.031** | N | X | | | | |
| **Clinical Judgment** | 0.375 | 0.999 | N | 0.125 | X | | | |
| **Predict Sepsis tool 1 ≥2** | 0.999 | 0.625 | N | **0.021** | 0.375 | X | | |
| **Predict Sepsis tool 2 ≥2** | 0.999 | 0.625 | N | **0.021** | 0.375 | 0.999 | X | |
| **Predict Sepsis tool 3 ≥2** | N | N | N | N | N | N | N | X |

NEWS2=National Early Warning score 2, RETTS= Rapid Emergency Triage and Treatment System. N= Not possible to calculate since RETTS orange and Predict screening tool 3 identified all patients with septic shock and the variable in the column was therefore a constant, not allowing McNemar´s test.

P-values derived from McNemar´s test are presented in the table. Bold numbers of P-values indicate a significant difference between the models with respect to sensitivity for septic shock.

References:

1) Royal College of Physicians. National Early Warning Score (NEWS) 2- Standardising the assessment of acute-illness severity in the NHS, Updated report of a working party December 2017.

2) Widgren BR, Jourak M. Medical Emergency Triage and Treatment System (METTS): a new protocol in primary triage and secondary priority decision in emergency medicine. The Journal of emergency medicine. 2011.

3) Wallgren UM, Sjölin J, Järnbert-Pettersson H, Kurland L. The predictive value of variables measurable in the ambulance and the development of the Predict Sepsis screening tools: a prospective cohort study. Scandinavian journal of trauma, resuscitation and emergency medicine. 2020.
