# Supplementary material for: Performance of NEWS2, RETTS, clinical judgment and the Predict Sepsis screening tools with respect to identification of sepsis among ambulance patients with suspected infection: a prospective cohort study
Source: Scand J Trauma Resusc Emerg Med. 2021 Sep 30;29:144. doi: 10.1186/s13049-021-00958-3 (PMC8485465; doi:10.1186/s13049-021-00958-3)
Supplement: Supplementary file 10 — Additional file 10. DeLong's test, pairwise comparison of AUC for septic shock for models with specific cut-offs. [file 13049_2021_958_MOESM10_ESM.pdf]

Additional file 10. DeLong´s test, pairwise comparison of AUC for septic shock for models with specific cut-offs.

Compared with:	**NEWS2[1] ≥5**	**NEWS2 ≥7**	**RETTS[2] ≥orange**	**RETTS red**	**Clinical Judgment**	**Predict Sepsis tool 1[3] ≥2**	**Predict Sepsis tool 2[3] ≥2**	**Predict Sepsis tool 3[3] ≥2**
**NEWS2 ≥5**	X							
**NEWS2 ≥7**	0.651	X						
**RETTS ≥orange**	0.738	0.547	X					
**RETTS red**	0.753	0.985	0.621	X				
**Clinical Judgment**	0.222	0.436	0.097	0.398	X			
**Predict Sepsis tool 1 ≥2**	**0.049**	0.072	**0.020**	0.143	**0.008**	X		
**Predict Sepsis tool 2 ≥2**	0.133	0.146	0.088	0.230	**0.021**	**0.023**	X	
**Predict Sepsis tool 3 ≥2**	0.057	0.103	**0.001**	0.188	**0.010**	0.361	0.794	X

NEWS2=National Early Warning score 2, RETTS= Rapid Emergency Triage and Treatment System.

P-values derived from DeLong´s test are presented in the table. Bold numbers of P-values indicate a significant difference between the AUC values (for models with specific cut-offs) with respect to outcome septic shock.

References:

1) Royal College of Physicians. National Early Warning Score (NEWS) 2- Standardising the assessment of acute-illness severity in the NHS, Updated report of a working party December 2017.
2) Widgren BR, Jourak M. Medical Emergency Triage and Treatment System (METTS): a new protocol in primary triage and secondary priority decision in emergency medicine. The Journal of emergency medicine. 2011.
3) Wallgren UM, Sjölin J, Järnbert-Pettersson H, Kurland L. The predictive value of variables measurable in the ambulance and the development of the Predict Sepsis screening tools: a prospective cohort study. Scandinavian journal of trauma, resuscitation and emergency medicine. 2020.
