# Supplementary material for: Performance of NEWS2, RETTS, clinical judgment and the Predict Sepsis screening tools with respect to identification of sepsis among ambulance patients with suspected infection: a prospective cohort study
Source: Scand J Trauma Resusc Emerg Med. 2021 Sep 30;29:144. doi: 10.1186/s13049-021-00958-3 (PMC8485465; doi:10.1186/s13049-021-00958-3)
Supplement: Supplementary file 11 — Additional file 11. ROC curves for models without cut-offs and septic shock. [file 13049_2021_958_MOESM11_ESM.pdf]

Additional file 11. ROC curves for models without cut-offs and septic shock.

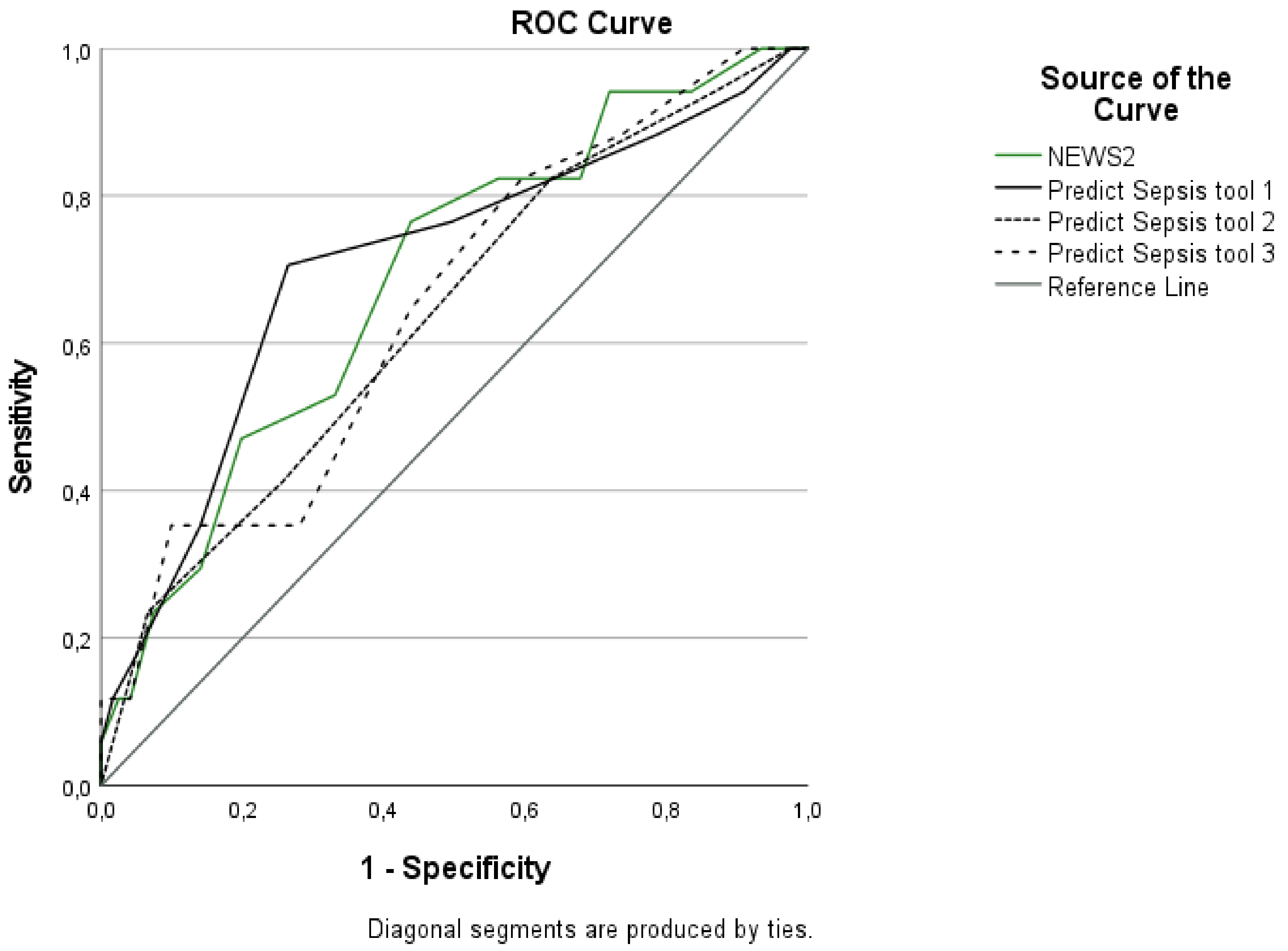

ROC=Receiver Operating Characteristic Curve, NEWS2=National Early Warning Score 2, RETTS= Rapid Emergency Triage and Treatment System
